# Journal of Automatic Chemistry: Editorial Board

**DOI:** 10.1155/S1463924690000013

**Published:** 1990


Journal of Automatic Chemistry Vol. 12, No. 1 (January–February 1990), p. 1

### Journal of Automatic Chemistry: Editorial Board

The journal's editorial board was substantially changed at the beginning of 1990 in order to increase *JAC*'s international and topical coverage. Thus the Editor welcomes the following 'new blood' members: D. J. Curran, University of Massachusetts at Amherst; S. R. Bysouth, University of Massachusetts at Amherst; G. A. Gibbon, US Department of Energy; H. M. (Skip) Kingston, NIST; G. Knapp, University of Technology, Graz; G. Michel, Ciba-Geigy AG; C. Vandecasteele, Gent University; and A. P. Wade, University of British Columbia. The journal will be publishing short biographies of the new members in the early issues of 1990—the aim is to introduce our various experts to readers and potential authors. Those of Drs Kingston, Wade and Vandecasteele follow.

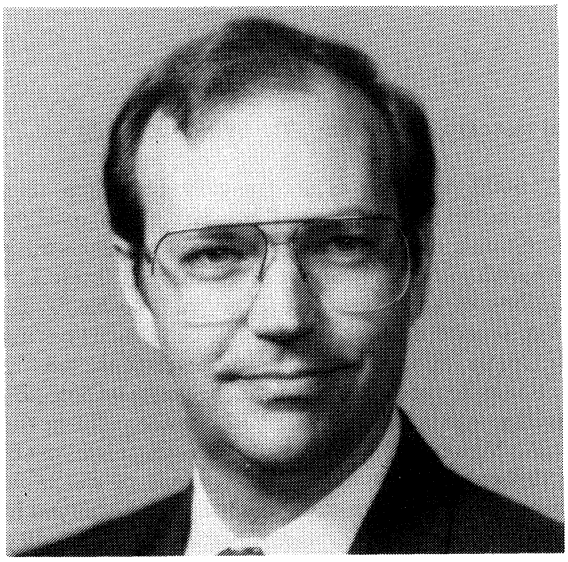

H. M. (Skip) Kingston is a Supervisory Research Chemist and Project Manager for the Consortium on Automated Analytical Laboratory Systems in the Inorganic Analytical Research Division at the National Institute of Standards and Technology (NIST) (formerly the National Bureau of Standards) in Gaithersburg, Maryland, USA. He received his B.S. and M.S. degrees in chemical education and analytical chemistry from Indiana University of Pennsylvania in 1973 and 1975 and his Ph.D. in analytical chemistry and environmental management from the American University in 1978. He is active in such standards organizations as the ASTM and IUPAC. He has pursued methods development in trace element analysis and developed chromatographic methods for matching sample form to instrument capability. He has participated in over 100 elemental certifications at NIST. His interests have included environmental analysis, laboratory robotics, microwave sample preparations, nuclear waste testing, and the future direction of automation for the analytical laboratory.

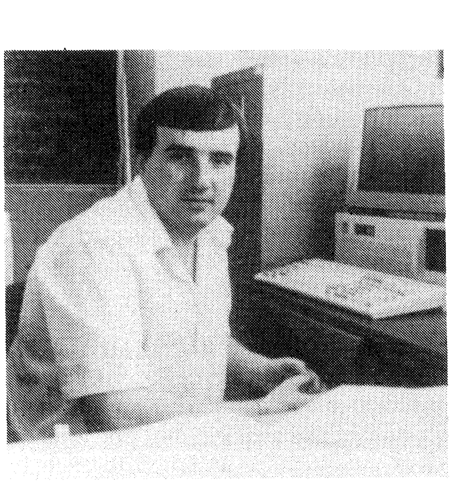

Adrian P. Wade received his B.Sc. in Chemistry with Computer Studies from The University of Southampton in 1981. In 1985 he obtained his Ph.D. in Chemistry ('Modern Mathematical Methods in Analytical Chemistry') from The University of Wales, under the supervision of Professor D. Betteridge. He was a Chemist/Computer Scientist from the British Petroleum Research Centre, Sunbury-on-Thames, UK from March 1985 to February 1987, and from September 1985 to June 1987 was based at Michigan State University, where, as a Visiting Research Associate, he worked on artificial intelligence for instrumental analysis. On joining the University of British Columbia in Vancouver in July 1987, Dr Wade founded the Laboratory for Automated Chemical Analysis and has since established research initiatives in chemical acoustic emission and automated flow injection analysis. This has required the development of novel automated (microcomputer-controlled) analytical instrumentation, and of new chemometric methods for automated analysis of multivariate data. The work has included several fruitful industrial collaborations.

Dr Wade has so far published 35 referred papers and a book chapter in the areas mentioned above. He is a Member of the Royal Society of Chemistry and of the Chemical Institute of Canada. He has recently been elected a Fellow of the Institution of Analysts and Programmers. In addition to his new association with the *Journal of Automatic Chemistry*, he is a board member and contributing editor for *Trends in Analytical Chemistry*, and is a member of the editorial advisory board for *Chemometrics and Intelligent Laboratory Systems.*

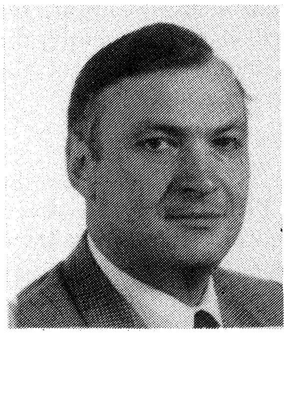

Carlo Vandecasteele obtained his Ph.D. in Analytical Chemistry from Gent University in 1975 and his 'Aggregaat Hoger Onderwijs' in 1984. He is a Research Director of the National Fund for Scientific Research (Belgium) at the Laboratory for Analytical Chemistry, Gent University, Gent, Belgium. Until 1986 his research interests were activation analysis, mainly with charged particles and production of radionuclides by means of a cyclotron. He developed automated procedures for the production of highly radioactive isotopes and labeled compounds. His current research relates to atomic spectrometry (ICP-MS, ICP-AES, AAS and AFS). In the field of optical spectrometry his interests include computerized data treatment and automation. He is author or co-author of several books and of some 150 scientific papers.

